# Publisher Correction: Imaging the square of the correlated two-electron wave function of a hydrogen molecule

**DOI:** 10.1038/s41467-018-04740-5

**Published:** 2018-06-05

**Authors:** M. Waitz, R. Y. Bello, D. Metz, J. Lower, F. Trinter, C. Schober, M. Keiling, U. Lenz, M. Pitzer, K. Mertens, M. Martins, J. Viefhaus, S. Klumpp, T. Weber, L. Ph. H. Schmidt, J. B. Williams, M. S. Schöffler, V. V. Serov, A. S. Kheifets, L. Argenti, A. Palacios, F. Martín, T. Jahnke, R. Dörner

**Affiliations:** 1 Institut für Kernphysik, J. W. Goethe Universität, Max-von-Laue-Str. 1, 60438 Frankfurt, Germany; 2 Departamento de Química, Universidad Autónoma de Madrid, 28049 Madrid, Spain; 3 Universität Kassel, Heinr.-Plett-Strasse 40, 34132 Kassel, Germany; 4 Institut für Experimentalphysik, Universität Hamburg, Luruper Chaussee 149, 22761 Hamburg, Germany; 5 FS-PE, Deutsches Elektronen-Synchrotron DESY, Notkestrasse 85, 22607 Hamburg, Germany; 6 FS-FLASH-D, Deutsches Elektronen-Synchrotron DESY, Notkestrasse 85, 22607 Hamburg, Germany; 7 Chemical Sciences Division, Lawrence Berkeley National Laboratory, Berkeley, CA 94720 USA; 8 Department of Physics, University of Nevada Reno, 1664 N. Virginia Street, Reno, NV 89557 USA; 9 Department of Theoretical Physics, Saratov State University, 83 Astrakhanskaya, Saratov, 410012 Russia; 10 Research School of Physical Sciences, The Australian National University, Canberra, ACT 0200 Australia; 11 Instituto Madrileo de Estudios Avanzados en Nanociencia, 28049 Madrid, Spain; 12 Condensed Matter Physics Center (IFIMAC), Universidad Autónoma de Madrid, 28049 Madrid, Spain; 13 Present Address: Department of Physics and CREOL College of Optics & Photonics, University of Central Florida, Orlando, FL 32816 USA

Correction to: *Nature Communications* 10.1038/s41467-017-02437-9, published online 22 December 2017

The original version of this Article contained an error in the fifth sentence of the first paragraph of the ‘Application on H_2_’ section of the Results, which incorrectly read ‘The role of electron correlation is quite apparent in this presentation: Fig. 1a is empty for the uncorrelated Hartree–Fock wave function, since projection of the latter wave function onto the 2*pσ*_u_ orbital is exactly zero, while this is not the case for the fully correlated wave function (Fig. 1d); also, Fig. 1b, c for the uncorrelated description is identical, while Fig. 1e, f for the correlated case is significantly different.’ The correct version replaces ‘Fig. 1e, f’ with ‘Fig. 2e, f’.

This has been corrected in both the PDF and HTML versions of the Article.

